# The Harold Amos Medical Faculty Development Program: Evaluation of a National Program to Promote Faculty Diversity and Health Equity

**DOI:** 10.1089/heq.2016.0022

**Published:** 2018-03-01

**Authors:** James P. Guevara, Melissa Wright, Nancy W. Fishman, David M. Krol, Jerry Johnson

**Affiliations:** ^1^ Department of Pediatrics, Perelman School of Medicine, University of Pennsylvania, Philadelphia, Pennsylvania.; ^2^ PolicyLab: Center to Bridge Research, Practice, and Policy, The Children's Hospital of Philadelphia, Philadelphia, Pennsylvania.; ^3^ Robert Wood Johnson Foundation, Princeton, New Jersey.; ^4^ Division of Geriatric Medicine, Perelman School of Medicine, The University of Pennsylvania, Philadelphia, Pennsylvania.

**Keywords:** faculty, healthcare disparities, medical, minority groups, program evaluation

## Abstract

**Purpose:** The Harold Amos Medical Faculty Development Program (AMFDP), a national program of the Robert Wood Johnson Foundation, seeks to support academic physicians from historically disadvantaged backgrounds and serves as a model program for promoting faculty diversity and health equity. Our objective was to determine differences in scientific productivity, promotions and retentions, and leadership attainment among faculty applicants to this national minority faculty development program.

**Methods:** Final-round interview applicants from 2003 to 2008 were selected. Differences in publications, grants, promotions/retentions, and leadership positions through 2013 were compared between funded scholars and unfunded nonscholars. Semistructured interviews were conducted to identify factors that facilitated and hindered academic success.

**Results:** A total of 124 applicants (76 scholars and 48 nonscholars) who participated in final-round interviews from 2003 to 2008 were eligible. Scholars and nonscholars had similar number of publications. Scholars had greater number of grants and grant dollars, but differences were not significant after accounting for AMFDP program awards. Scholars were more likely to hold leadership positions (28% vs. 10%, *p*=0.02), but equally likely to be promoted (67% vs. 58%, *p*=0.32) and retained (84% vs. 75%, *p*=0.21). In interviews, all participants endorsed mentoring, funding, and nonscientific education to academic success, but scholars reported greater availability of leadership opportunities consequent to AMFDP.

**Conclusion:** There were few differences in academic productivity attributable to a national faculty diversity program. However, program participants were more likely to endorse and attain leadership positions. Academic institutions should consider facilitating leadership development of minority faculty as a means of advancing health equity research and training.

## Introduction

Despite recent efforts, health inequities among U.S. population subgroups continue to persist.^1,2^ An important strategy to reducing health inequities is to build institutional capacity to conduct health disparities' research and training.^3^ Minority physician scientists play key roles in setting institutional agendas, expanding research, and serving as role models for ameliorating health inequities.^4^ As a result, the Institute of Medicine, now the National Academy of Medicine, has advocated for greater faculty diversity.^5^

Underrepresented in medicine (UIM) physicians consist of individuals from racial and ethnic populations that are underrepresented in the medical profession relative to their numbers in the general population.^6,7^ UIM physicians comprise just 9% of medical school faculty compared with 18% of medical students and 35% of the U.S. population.^7,8^ Although UIM representation in academia has increased over time,^9^ UIM physicians are less likely to be promoted than their majority peers,^10–13^ to hold administrative leadership positions,^12,14,15^ and to receive National Institutes of Health (NIH) research awards.^16^ UIM faculty report lower career satisfaction and social isolation, leading to greater attrition than nonminority faculty.^17–20^ The reasons for this underrepresentation are multifactorial, but likely include bias and a lack of appropriate mentorship.^10,16^

Minority faculty development programs have been developed at institutions of higher education to foster minority faculty career development. One such program is the Harold Amos Medical Faculty Development Program (AMFDP), a national postdoctoral award program of the Robert Wood Johnson Foundation (RWJF) since 1983. AMFDP has sought to increase the number of medical school faculty from historically disadvantaged backgrounds who achieve senior academic rank.^21,22^

It is not clear to what extent AMFDP and similar programs benefit UIM physician scientists. The AMFDP has a highly competitive application process, seeks applicants from research-intensive institutions with availability of resources, and draws a high caliber of applicants applying to the program. The objective of this study was to determine differences in academic productivity, promotion, retention, and attainment of leadership among a cohort of former AMFDP-funded scholars and unfunded nonscholar applicants. Information from this study can be used to improve minority faculty development at academic institutions and contribute to institutional capacity to reduce health inequities.

## Methods

### Study participants

Final-round AMFDP applicants from 2003 to 2008 were selected for inclusion in this study. Scholars were defined as individuals who applied to AMFDP during the 2003–2008 application period, completed final-round interviews and were subsequently funded, regardless of whether they completed the program or not. Nonscholars were defined as individuals who applied to AMFDP during the 2003–2008 application period and completed final-round interviews, but were not funded. Identifying information on scholars and nonscholars was obtained from computerized files of AMFDP's National Program Office. The study was restricted to the above years, as applicant data before 2003 was not available, and scholars from 2009 and beyond had not completed the program at the time of study initiation. The study received an exemption from review by the Institutional Review Board at the Children's Hospital of Philadelphia.

### Measures

We obtained demographic information (age, gender, race/ethnicity, rank, application year, institution, and contact information) on scholars and nonscholars from the AMFDP's National Program Office. For each scholar and nonscholar, we completed searches of the following electronic databases from the year of their application through December 2013: Medline, HealthStar, Biosis Previews, CINAHL, PsychINFO, Environment Abstracts, Global Health, and Scopus. We sought to identify publications in the basic sciences, translational science, epidemiology or public health, clinical medicine, and health services research. The full text of all abstracts with corresponding name matches to a scholar or nonscholar was obtained, and corresponding names and current and past institutional affiliations were checked to verify identities. Articles that were unable to be verified were queried for confirmation directly from study participants at the time of interviews. Articles were categorized as peer-reviewed scientific publications or nonpeer-reviewed articles, which included letters, editorials, reviews, and book chapters. We utilized Scopus to determine each participant's H-Index, a measure of the overall level of citations attributed to an individual's publications.^23^

In addition, we completed searches of the following electronic grant databases from the year of their application through December 2013: the NIH reporter, a database of federal grant awards, including NIH, Health Resources and Services Administration (HRSA), Agency for Healthcare Research and Quality, Centers for Disease Control and Prevention, and Food and Drug Administration; the website of the RWJF; the website of the Pew Charitable Trusts; the website of the Commonwealth Fund; and the website of the Annie E. Casey Foundation. We sought to identify scholars and nonscholars who were listed as a principal investigator on any independent grant awards. Corresponding names and current and past institutional affiliations were checked to verify identities. Awards that were unable to be verified were queried for confirmation directly from study participants at the time of interviews.

To verify and supplement information obtained from database searches, we conducted Google searches of all scholars and nonscholars by name. We sought curriculum vitae directly from study participants at the time of interviews or from on-line sources at institutional websites. We defined promotion as at least a one-level increase in academic rank from the time of application through December 2013. We defined retention as maintaining an academic affiliation with a U.S. medical school at the time of the evaluation. We considered leadership positions in the following categories: administrative (e.g., division chief or department chair), clinical (e.g., medical director), research (e.g., research center or institute director), teaching (e.g., fellowship, program, or clerkship director), and professional (e.g., society president).

### Semistructured interviews

To identify barriers and facilitators to academic success among minority faculty, we conducted semistructured telephone interviews with scholars and nonscholars. We contacted participants using letters of introduction from the AMFDP's National Program Office and scheduled interviews using email and telephone solicitation. We developed an interview guide based in part on a previous survey study and pilot tested questions with minority faculty at the Perelman School of Medicine at Penn (see interview guide in the Supplementary Data).^24^ Questions queried participants regarding mentoring experiences, educational opportunities, and funding and how these affected their career advancement following their application to AMFDP. At the time of interviews, we verbally consented participants, audiorecorded interviews, and transcribed interviews for coding and analysis. We provided participants with a $100 study incentive for participation.

### Analysis

To determine differences in scientific productivity, we assessed differences in mean publications (total, peer reviewed, and nonpeer reviewed), mean grants (total, federal, and foundation), mean grant dollars (total, federal, and foundation), and H-Index scores between scholars and nonscholars using *t*-tests and multiple regression models adjusting for age, gender, race/ethnicity, application year, change of institutions, and rank at the time of application. To determine differences in promotion, retention, and leadership, we assessed differences in the proportion who were promoted (an increase of one rank or higher), proportion who achieved leadership positions (any, clinical, research, teaching, administrative, and professional), and proportion who remained in academic medicine using chi-square tests and multiple logistic regression models adjusting for age, gender, race/ethnicity, application year, and rank at the time of application.

All interview transcripts were read by at least two individuals (J.G. and M.W.). Codes were developed among the investigative team using an iterative consensus process and applied to transcripts. Themes relating to mentorship, educational opportunities, and funding from a previously developed conceptual framework were identified as barriers or facilitators to academic success using an inductive process.^24^

## Results

We identified 76 scholars and 48 nonscholars who applied and were interviewed for AMFDP from 2003 to 2008. Scholars and nonscholars were similar with respect to age, gender, and rank at the time of application, but scholars were more likely to be Hispanic than nonscholars (Table 1). At the time of application, 44 (35%) were still trainees, including residents and fellows, 28 (23%) had provisional faculty appointments, including instructor, and 52 (42%) were assistant professors.

**Table 1.** **Demographic Characteristics of Scholars and Nonscholars**

Characteristic	Scholar (*n*=76)	Nonscholar (*n*=48)
Mean age (SD)	45.4 (3.5)	44.2 (3.9)
Gender, *n* (%)		
Male	45 (59)	20 (42)
Female	31 (41)	28 (58)
Race, *n* (%)^a^		
African American	52 (68)	39 (81)
Hispanic Latino	23 (30)	6 (13)
Native American	1 (1)	3 (6)
Cohort, *n* (%)		
2003	11 (15)	7 (15)
2004	10 (13)	7 (15)
2005	14 (18)	9 (19)
2006	14 (18)	6 (13)
2007	13 (17)	12 (25)
2008	14 (18)	7 (15)
Rank at T_0_, *n* (%)		
Resident	2 (3)	0 (0)
Fellow	24 (32)	18 (38)
Instructor	12 (16)	12 (24)
Assistant professor	35 (45)	17 (35)
Other	3 (4)	1 (3)

Final-round applicants to the Harold Amos Medical Faculty Development Award included funded scholars and nonscholars without funding.

^a^ Difference between scholars and nonscholars is statistically significant, *p*<0.05.

SD, standard deviation.

Scholars and nonscholars had similar levels of academic productivity (Table 2). Scholars and nonscholars were not different with respect to publications and H-index scores. Scholars had a greater mean number of total grants and grant dollars than nonscholars. There were no differences in federal grant awards or federal grant dollars. Differences in adjusted mean publications, H-Index scores, total grants, and total grant dollars were similar to the unadjusted results. However, differences in mean total grant dollars between scholars and nonscholars were no longer statistically significant after adjustment. In addition, differences in mean number of grants were no longer statistically significant after adjusting for the AMFDP award.

**Table 2.** **Academic Productivity of Harold Amos Scholars and Nonscholars**

Productivity measure	Scholar (*n*=76)	Nonscholar (*n*=48)	*p*
Mean publications (SD)			
Total	27.2 (30.0)	33.0 (58.8)	0.47
Peer reviewed	19.5 (20.3)	24.4 (41.9)	0.39
Nonpeer reviewed	7.7 (12.0)	8.9 (20.1)	0.71
Mean grants (SD)			
Total	2.0 (1.5)	0.7 (1.0)	<0.001
Federal	1.0 (1.5)	0.7 (0.9)	0.19
Foundation	1.0 (0.2)	0.1 (0.2)	<0.001
Mean grant dollars (SD)			
Total in thousands	1463 (2390)	567 (1507)	0.02
Federal in thousands	1049 (2372)	560 (1509)	0.21
Foundation in thousands	385 (60)	7 (41)	<0.001
H-Index (SD)	12.5 (7.9)	10.9 (10.2)	0.32

Final-round applicants to the Harold Amos Medical Faculty Development Award included funded scholars and nonscholars without funding.

A greater percentage of scholars were promoted in rank (67% vs. 58%, *p*=0.32) and retained in an academic position (84% vs. 75%, *p*=0.21) than nonscholars, but these differences were not statistically significant (Table 3). Twenty scholars (26%) and seven nonscholars (15%) moved institutions following application (*p*=0.12); individuals who moved institutions were more likely to leave academic medicine (*p*=0.04), but were equally likely to be promoted (*p*=0.15). Similarly, a greater number of scholars than nonscholars achieved a rank of associate professor or greater (34% vs. 25%, *p*=0.35), but these results were similar after adjustment.

**Table 3.** **Promotion and Leadership Advancement of Harold Amos Scholars and Nonscholars**

	Scholar *n*=76, *n* (%)	Nonscholar *n*=48, *n* (%)	*p*
Overall promotion	51 (67.1)	28 (58.3)	0.32
Associate professor/professor	26 (34.2)	12 (25.0)	0.35
Any leadership position	21 (27.6)	5 (10.4)	0.02
Administrative	2 (2.6)	0 (0)	0.28
Research	11 (14.5)	2 (4.2)	0.07
Clinical	12 (15.8)	3 (6.3)	0.11
Teaching	0 (0)	1 (2.1)	0.21
Medical/professional	0 (0)	1 (2.1)	0.21
Retention	64 (84.2)	36 (75.0)	0.21

Final-round applicants to the Harold Amos Medical Faculty Development Award included funded scholars and nonscholars without funding.

Scholars were more likely to report attaining a leadership position (28% vs. 10%, *p*=0.02). Most of the differences in leadership positions were reported as either research (14.5% vs. 4.2%) or clinical (15.8% vs. 6.3%) positions. After adjustment, scholars were more likely to attain a leadership position (adjusted odds ratio [AOR] 3.9, 95% confidence interval [CI] 1.2–14.0) than nonscholars. In addition, scholars and nonscholars, who were at the assistant professor rank at the time of application, were more likely to attain leadership positions (AOR 3.6, 95% CI 1.0–12.9).

Of the 124 eligible participants, we completed interviews with 63 (51%). By application year, there were moderate differences in the number and proportion who completed interviews: 10 (56%) in 2003, 11 (65%) in 2004, 7 (30%) in 2005, 10 (50%) in 2006, 15 (60%) in 2007, and 10 (48%) in 2008. Scholars were more likely to complete interviews (52% vs. 48%, *p*<0.01) than nonscholars.

Scholars and nonscholars agreed on the importance of mentoring to facilitate career advancement and to help navigate the pathway to promotion (Table 4). Good mentors were perceived as being honest, available, encouraging, respectful, good at listening, and invested in their mentee's advancement; whereas bad mentors were perceived as lacking concern for the development of their mentees, unavailable, or taking advantage of their mentees to promote their own careers. Participants also emphasized the importance of networking and peer mentoring with other UIM faculty. Scholars uniformly reported that national mentors and program staff helped facilitate leadership opportunities, but this was not reported to a similar degree among nonscholars. However, both groups perceived the importance of leadership opportunities.

**Table 4.** **Scholar and Applicant Themes Concerning the Harold Amos Medical Faculty Development Program**

Theme	Exemplary quote
Goal of mentorship is to provide guidance on career advancement and navigating the promotion pathway	“Also, you know, at the same time, one who sort of knows the ropes, if you will, and has experience in sort of navigating the promotion pathway at [name].” (2003 Scholar)
Characteristics of good mentors include honesty, availability, encouragement, respectful, good listener, and invested in another's career	“It's much more of a relationship built on respect as well as one that focuses on my advancement.” (2003 Applicant)
Characteristics of bad mentors include lack of concern for development of others, lack of time, and taking advantage of others	“He's too busy. He's too famous. Usually I'm calling him and he is, I don't know, someplace else on the globe, not usually in my time zone…” (2004 Scholar)
Role of mentors is to help chart career paths, facilitate opportunity, provide guidance on projects, and understand individual strengths	“She's been a career sponsor, not just a mentor. So she's someone who's put my name in for talks. She's put my name in for leadership positions, for research connections.” (2004 Scholar)
Importance of discerning institutional climate and potential for bias and how to deal with it	“I think they probably feel more comfortable with a mentor that looks like them and has the same–that may have the same issues or problems that need to be overcome and also as a perspective on how to be successful.” (2006 Scholar)
Need to be self-taught in certain areas due to lack of educational opportunities or personal preferences	You learn best, I think, when you write a grant and other people then critique it and say well what about this, what about this, where can you put this, you know, that type of stuff.” (2003 Applicant)
	“I think that something that I wish I would have maybe learned or gotten more mentoring on earlier on is kind of some of the interpersonal stuff about networking and how you present yourself in meetings and things like that. So some of that I think might have been helpful earlier on.” (2004 Scholar)
Mentoring is important but there are few opportunities to formally learn it	“I think you draw from other people. So there's an old saying in some of those surgical programs, you learn from peoples mistakes, not just your own you have to learn from other people's mistakes. So a lot of my mentorship was drawn from what I felt I was lacking in my own mentorship.” (2003 Applicant)
Peer networks have great potential but are underutilized	“[People] could benefit from a network of people that are able to review publications and/or grants.” (2004 Scholar)
Need to be open to respond to opportunities and challenges when they arise	“I was working in Haiti and there was a major earthquake, as you probably know. So there was a lot of need, actually. So my career actually took a different turn after that…” (2007 Scholar)
Lack of diversity is difficult to discuss, creates isolation, and forces people to overwork to be recognized	“… when there are not enough faculty of color, there are just more demands that are made on your time. And both–you want to be able to do a lot more than maybe you should. And I think it's natural that if people–and it's really you in respect to your opinion. They are asking you to participate. And when you're–when you have been given the opportunity to be at the table and be a part of conversations, you're very hesitant to say no.” (2006 applicant)
Poor funding climate can push you out of academia, but funding can facilitate an academic career	“But that said, if you don't have funds, nothing. So you really, really cannot overestimate or overstate the importance of getting a career award and not just any career award, but a prestigious one at that, that makes waves on your résumé, gives you access to people, as well as from an organization.” (2008 Scholar)
AMFDP is highly valued for quality, networking, skills taught, and encouragement	“I mean just the focus on just high quality, very rigorous, you know, just critical thinking, that part of it, merged with a network of people who are just committed, and you just don't find that often, I mean people who are really, really committed to your success, and allow themselves to be accessed.” (2005 Scholar)
AMFDP should provide feedback to applicants to improve future applications	“I think that the Robert Wood Johnson was definitely something that I was not successful. … But because of the specific goal of this granting agency to increase the pipeline of physicians of color, I think it actually would be helpful if feedback could be given to applicants…” (2006 Applicant)
Main benefit of AMFDP was provision of unique opportunities for advancement	“I think it opened up completely different collaborations that I didn't have access to.” (2007 Scholar)
	“The Harold Amos program, I will remain indebted to it. Particularly for someone of my background, even someone–even if you were born here with all the privileges, you feel indebted to it, because it's nice to be part of something, it's nice to have something that gives you negotiating tool…” (2008 Scholar)

Final-round applicants to the Harold Amos Medical Faculty Development Award were either selected as scholars and received funding or nonscholars without funding.

AMFDP, Harold Amos Medical Faculty Development Program.

Scholars and nonscholars reported on the availability of courses and workshops that were designed to enhance their scientific skills and advance their careers, for example, grant writing or article preparation (Table 4). However, they reported a dearth of nonscientific courses in areas they perceived to be important, for example, negotiation, grants management, leadership, and work-life balance. They perceived a need to be self-taught in these areas. In particular, they reported a lack of training in mentoring, despite the fact that they universally reported mentoring trainees from undergraduates through postdoctoral fellows.

Regarding social climate, scholars and nonscholars perceived a lack of diversity among faculty across institutions (Table 4). This lack of diversity was perceived by participants to be difficult to discuss openly among their peers and supervisors, to create a sense of isolation, and to lead to feelings of being under-recognized for one's accomplishments. As a result, both scholars and nonscholars voiced the importance of understanding an institution's social climate to be successful, the potential for bias that may exist at academic institutions, and the need for receiving advice on how to deal with a lack of diversity and potential unconscious bias. There were differences among participants as to the need to have a mentor of color. Some felt it important to receive advice from a mentor who had personally struggled with and overcame bias, whereas others felt that it was sufficient to have a mentor of any color who understood the reality of unconscious bias and could provide relevant advice.

All participants acknowledged the importance of garnering funding to advance their academic careers (Table 4). Those who changed career paths reported a lack of sufficient grant funding necessitating a change in their career direction. For this reason, some perceived the need to be open to challenges and opportunities when they arose and to be willing to take their career in a different direction than the one they had previously anticipated.

## Discussion

In this evaluation of AMFDP, a national minority faculty development program of the RWJF, we compared a 6-year cohort of funded scholars and unfunded nonscholars on measures of academic productivity, promotion, retention, and leadership. We found few differences in academic productivity, promotion, and retention, suggesting that individuals who are selected for final-round interviews for AMFDP are similarly talented and driven to succeed. However, we found that scholars were more likely to report attainment of leadership positions. This suggests that participation in AMFDP provides unique leadership opportunities that may not be ordinarily available to talented UIM faculty at research-intensive institutions. Indeed, our interviews confirmed that national mentors and program staff help facilitate opportunities for scholars to advance in their careers. In addition, networking sessions during national AMFDP meetings with fellow scholars and national advisory committee members were reported to provide additional opportunities for career advancement than what was available at their home institutions.

Our results are similar to a previous AMFDP evaluation, which found that 88% of former scholars remained in academic medicine and 33% were promoted to associate professor rank or higher.^21,22^ In our evaluation, we found that 84% of scholars in a 6-year cohort remained in academic medicine and 34% had been promoted to associate professor or higher (23 to associate professor and 3 to professor).

Our results differ from recent national studies of UIM promotion at academic medical centers. Nunez-Smith et al.^25^ reported an institutional median of 24% of Hispanic and 19% of black faculty who were promoted to associate professor, compared to 30% of white faculty. We previously reported a lack of association between minority faculty development programs and promotion of UIM faculty.^9^ Our results from the current study, however, suggest that participants in the AMFDP may be more likely to be promoted than other minority faculty at large academic medical centers, but results were not statistically significant given the small sample size.

The results of our interviews confirmed the importance of mentoring, nonscientific educational opportunities, and funding for career advancement in academia. This is consistent with the results from HRSA-funded Centers of Excellence.^26–29^ The AMFDP was well regarded by scholars and nonscholars alike for its ability to provide these tools and resources for UIM faculty to succeed. In particular, scholars commented on the importance of networking available through AMFDP with fellow scholars and individuals of national prominence who could provide guidance, opportunity, and encouragement.

The results with regards to leadership are of importance. Pololi et al.^30^ surveyed academic faculty at 26 medical schools and reported greater leadership aspirations among UIM faculty than non-UIM faculty. Our results are consistent with this study. We found that scholars in AFMDP were more likely to attain leadership positions in academic medicine, particularly in research and clinical areas, than nonscholars. AMFDP has set as one of its goals to have scholars achieve senior faculty and leadership positions and offers scholars networking opportunities with individuals of national prominence at its national meetings and assigns national mentors who can facilitate leadership positions. However, our interviews suggest that training in structured or systematic leadership development is lacking and may be an important focus for programs targeting UIM faculty.

Our finding that scholars were not more academically productive than nonscholars should not be surprising, given the talent pool among those applicants who were invited for a final interview for AMFDP. Although scholars were more productive with receipt of grants, this difference was no longer significant when the AMFDP grant was excluded. Applicants in our analysis had a mean of 33 publications, 1 federal grant, and an H-Index of 10.9, all very impressive numbers for junior faculty. The ability of AMFDP and similar programs to alter scholars' academic productivity may be limited given the high trajectory they and nonscholar final-round applicants already have achieved. In addition, scholars and nonscholars alike reported that their home institutions provided workshops and training in scientific domains, relevant to academic productivity, for example, grant writing, or article development. What scholars reported in interviews as lacking was the absence of training in nonscientific areas, for example, negotiation skills.

Scholars and nonscholars reported perceptions of unconscious bias at their home institutions and a lack of understanding with how to approach it. This is consistent with a previously published work by HRSA-funded Centers of Excellence.^26–29^ While it may not be possible to alter institutional bias through a program such as AMFDP, it may be possible to provide scholars with tools and resources to address it, such as through mentorship with senior faculty who are aware of it and have experience addressing it.

There are limitations with regard to our study. First, although we evaluated a 6-year cohort of scholars and applicants, our sample size of 124 may have been underpowered to find statistically significant differences on measures of promotion and retention. Second, our study evaluated scholars who were at most 7 years and at a minimum of 2 years following completion of AMFDP program. This time was not sufficient for all participants to have the opportunity to attain senior faculty ranks and leadership positions. Third, only 51% of scholars and applicants responded to our requests and completed interviews. However, our response rate is similar to another published survey we conducted involving physicians.^31^ Fourth, nonscholars may have received informal advice and mentoring as part of the application process, which may have assisted their careers and moved the results toward the null. Fifth, this evaluation of the AMFDP compared UIM faculty at research-intensive institutions. The majority of UIM faculty reside at nonresearch-intensive institutions.^9^ Sixth, over half of applicants (55%) were at the level of instructor or below at the time of application. Promotion to assistant professor at some institutions is less dependent on academic productivity and more dependent on completion of training and board certification.

### Health equity implications

The findings of this evaluation of the AMFDP have implications for research-intensive academic institutions that seek to advance research and training in health equity. First, well-developed and comprehensive minority faculty development programs like AMFDP can have a positive impact on the promotion and retention of UIM faculty. Second, given the perceived value of mentoring, scientific career development, and funding, institutions should ensure that these programs are readily available to UIM faculty. However, additional consideration should be given to providing training and skills development in nonscientific areas, particularly in leadership development, as UIM faculty perceive these skills as important to career advancement. Third, institutions should facilitate opportunities for leadership, as program scholars were more likely to attain leadership positions. Having more UIM faculty in leadership roles may increase the visibility of UIM faculty, enhance institutional reputation, reduce implicit bias, and expand diversity of viewpoints in institutional decision making. Fourth, institutions should track relevant metrics, including promotion and retention to ensure that their programs are meeting the needs of UIM faculty.

## Supplementary Material

Supplemental data

## Abbreviations Used

AMFDP: Harold Amos Medical Faculty Development Program
AOR: adjusted odds ratio
CI: confidence interval
HRSA: Health Resources and Services Administration
NIH: National Institutes of Health
RWJF: Robert Wood Johnson Foundation
SD: standard deviation
UIM: underrepresented in medicine
