# Supplementary material for: The Harold Amos Medical Faculty Development Program: Evaluation of a National Program to Promote Faculty Diversity and Health Equity
Source: Health Equity. 2018 Mar 1;2(1):7–14. doi: 10.1089/heq.2016.0022 (PMC6071893; doi:10.1089/heq.2016.0022)
Supplement: Supplemental data [file Supp_Data.pdf]

Harold Amos Medical Faculty Development Program Evaluation
Interview Guide

Hello, I'm calling from The Children's Hospital of Philadelphia. My name is Melissa. We are conducting a study funded by the Robert Wood Johnson Foundation to evaluate the Harold Amos Medical Faculty Development Program. Your name and contact information were provided by the Foundation as someone who applied to be in the faculty development program, and I'd like to ask you a few questions related to your experience with the program and your activities since your application. The interview consists of 24 questions and will take approximately 30-45 minutes to complete. You will be compensated $50 for your time. You are free to answer questions as you see fit and are under no obligation to answer any questions you choose not to. You are free to withdraw from the interview at any time. As with any study involving the collection of data, there is a risk of disclosure of your responses and other information collected. Every precaution will be taken to protect your personal information and ensure confidentiality. Risks will be minimized by storing identifiable data on secure servers and password protected files. You will not be identified in any publication resulting from this study. You may not benefit directly from the study, but you will contribute directly to improving the Harold Amos Medical Faculty Development Program and generating new knowledge. With your permission, I will audiotape and transcribe the interview. Do you have any questions?

1. To confirm, you applied and interviewed for the Harold Amos Faculty Development Program in ***(Year(s)***. (Affirm acceptance/non acceptance status if brought up by study subject but need for their invaluable perspective.)

2. First, I'd like to talk about mentoring that you may have received in the years after completing your application. I am interested in mentoring you may have received as a result of your association with the faculty development program or through other means. An example of mentoring would be 1:1 mentoring to enable individuals to solicit advice, facilitate opportunities, and receive support on a variety of topics including career advancement, scholar development, and work-life balance. Have you received any such mentoring since **(start year)**? ***(If no, probe as to why not, then skip to question #5)***

3. During this time, what were the names of your mentors? How did you identify them as a mentor?

4. Can you tell me more about each mentoring relationship? **Go through each mentoring relationship one by one**

   Probes:
   - Relationship with mentor
   - duration of relationship
   - frequency of meetings
   - topics discussed,
   - involvement Categories: scientific direction, collaboration, leadership, facilitation, navigating university systems, other)
   - What made **(X)** a good mentor. What made **(X)** a bad mentor
   - How did you benefit from mentorship?
   - What do you wish you received that you didn't?

5. Have you mentored anyone since **(start year)?** ***(If no, probe as to why not, then skip to question #8***

6. What are the names of the people you mentored?

7. Can you tell me more about the people you mentored?
    Probes
    - Relationship with mentor
    - duration of relationship,
    - frequency of meetings
    - topics discussed,
    - involvement Categories: scientific direction, collaboration, leadership, facilitation, navigating university systems, other)
    - where did you learn to be a mentor

8. The next set of questions will focus on career development since you completed your application. By career development, I am referring to research training or technical skill development that can enable faculty to hone skills necessary for promotion. An example of this would be participating in a career development course series designed to teach critical technical skills necessary for a successful academic career, such as grant writing or oral presentations. Have you participated in career development activities between since ***(start year)***? ***(If no, probe as to why not, then skip to question #11)***

9. During this time, what were the source(s) of career development?

10. Can you tell me more about each career development source? **Go through each career development source one by one**
    Probes
    - topics covered: teaching, manuscript writing; duration, formal program
    - How did you benefit from taking part in career development opportunities?
    - What do you wish you received that you didn't?

11. The next set of questions will focus on organizational climate. By organizational climate I am referring to the environment at your institution that affects morale, retention, and productivity and programs to enhance the educational and promotional climate for inclusion among the institution's faculty. An example would be the development of peer support groups that provide opportunities for scholars to network, collaborate, or otherwise support each other. Have you participated in activities aimed at inclusion since ***(start year)***? ***(If no, Probe as to why not, then skip to question 14)***

12. What are the source(s) of organizational climate opportunities?

13. Can you tell me about the social climate opportunities you participated in? **Go through each social climate source one by one**
    Probes
    - what did it consist of
    - frequency
    - How did you benefit from social climate opportunities?
    - What do you wish you received that you didn't?

14. The next set of questions will focus on financial awards you received since ***(start year).*** Financial awards correspond to monetary or equivalent aid that relieves faculty of clinical and/or administrative duties in order to pursue scholarly endeavors to advance their career or to obtain preliminary data for a subsequent award. A financial award may have also enabled you to attend a meeting or conference. An example of this is the provision of faculty start-up packages that provide time-limited salary support, protected time, and research support to enable faculty to establish research independence. Did you receive a financial award during this time period? ***(if no, skip to question # 17)***

15. What are your sources of financial awards?

16. Can you tell me more about the financial awards you received? **Go through each financial award source one by one**

    Probes:
    - Type of award
    - Source (federal, foundation, institution, other)
    - Salary support/percentage.
    - Purpose/aim of award,
    - Amount of award
    - duration of award
    - How did you benefit from financial packages? What did it enable you to do?
    - What do you wish you received that you didn't?

17. Now I'd like to ask you some questions about you have been doing since ***(start year).*** Where are you currently working?

18. What are your current rank, title, and department? If no longer in academic medicine, what happened to precipitate the change? Is this a full time position? If not a full time position, why did you drop down to part time, how long have you been in part time status?

19. Have you received any new academic or administrative appointments since **(start year)?** If yes, when and appointed to? If not, please describe your current position.

20. Have you been promoted since **(start year)?** If yes, when and promoted to?

21. Have you received any awards or been invited to join a professional society of distinction since ***(year)?*** If yes, which ones? (includes teaching awards, federal study sections, national scientific committees, leadership on professional societies)

22. What is your gender? (If not easily determined)

1. Lastly, to obtain more information about your achievements would you be willing to share your academic CV and grants pages?

Thank You. That was the last question. We really appreciate you taking part in this study. Goodbye.
